# Ultrasound super-resolution imaging for non-invasive assessment of microvessel in prostate lesion

**DOI:** 10.1186/s40644-024-00819-z

**Published:** 2025-01-07

**Authors:** Xin Huang, Huarong Ye, Yugang Hu, Yumeng Lei, Yi Tian, Xingyue Huang, Jun Zhang, Yao Zhang, Bin Gui, Qianhui Liu, Ge Zhang, Qing Deng

**Affiliations:** 1 Department of Ultrasound Imaging, Renmin Hospital of Wuhan University, Wuhan, 430060 China; 2 Department of Medical Ultrasound, China Resources & Wisco General Hospital, Wuhan University of Science and Technology, Wuhan, 430080 China; 3 Physics for Medicine Paris, Inserm U1273, ESPCI Paris, PSL University, Paris, 75015 France

**Keywords:** Prostate lesion, Ultasound super-resolution imaging, Microvessel, Non-invasive diagnosis

## Abstract

**Background:**

Prostate cancer (PCa) is the leading cause of cancer-related morbidity and mortality in men worldwide. An early and accurate diagnosis is crucial for effective treatment and prognosis. Traditional invasive procedures such as image-guided prostate biopsy often cause discomfort and complications, deterring some patients from undergoing these necessary tests. This study aimed to explore the feasibility and clinical value of using ultrasound super-resolution imaging (US SRI) for non-invasively assessing the microvessel characteristics of prostate lesion.

**Methods:**

This study included 127 patients with prostate lesion who presented at Renmin Hospital of Wuhan University between November 2023 and June 2024 were included in this study. All the patients underwent transrectal US (TRUS), contrast-enhanced US (CEUS), and US SRI. CEUS parameters of time-intensity curve (TIC): arrival time (AT), rising time (RT), time to peak (TTP), peak intensity (PKI), falling time (FT), mean transit time (MTT), ascending slope (AS), descending slope (DS), D/A slope ratio (SR), and area under the TIC (AUC). US SRI parameters: microvessel density (MVD), microvessel diameter (D), microvessel velocity (V), microvessel tortuosity (T), and fractal number (FN), were analyzed and compared between prostate benign and malignant lesion.

**Results:**

The tumor markers of prostate in the malignant group were all higher than those in the benign group, and the differences were statistically significant (*P* < 0.001). The TIC parameters of CEUS revealed that the PKI, AS, DS, and AUC were significantly higher in the malignant group than in the benign group (*P* < 0.001), whereas the RT, TTP and FT in the malignant group were significantly lower (*P* < 0.001). Malignant lesion exhibited significantly higher MVD, larger D, faster V, greater T, and more complex FN than benign lesion (*P* < 0.001).

**Conclusions:**

US SRI is a promising non-invasive imaging modality that can provide detailed microvessel characteristics of prostate lesion, offering an advancement in the differential diagnosis for prostate lesion. And, US SRI may be a valuable tool in clinical practice with its ability to display and quantify microvessel with high precision.

## Background

Prostate cancer (PCa) is the second most commonly diagnosed cancer in men worldwide, with rapidly increasing incidence and mortality rates, seriously threating to men’s health [1, 2]. Early diagnosis is crucial for effective treatment and prognosis of patients with PCa. Currently, pathological diagnosis is considered as the gold standard for diagnosis of PCa, and image-guided prostate biopsy is widely used in clinical practice [3]. The development of targeted biopsy techniques, particularly the use of fusion imaging for guided biopsy, has effectively reduced the number of needle insertions and improved diagnostic accuracy. These techniques mainly include magnetic resonance imaging (MRI) and positron emission tomography/computed tomography (PET/CT) combined with ultrasound (US) [4, 5]. However, the invasive nature of biopsy procedures can lead to discomfort and complications such as bleeding and infection, causing some patients to decline the procedure.

Owing to the limitations of invasive procedures, utilizing non-invasive examinations is essential for accurate diagnosis in PCa patients. Diagnosis of PCa can be associated with prostate microcirculation, and angiogenesis plays a momentous role in the local infiltration, growth, and distant metastasis of tumors [6]. Moreover, the microvessel morphology and functional characteristics differ between prostate benign and malignant lesion [7]. Therefore, assessing the microvessel distribution and flow characteristics within prostate lesion by non-invasive methods is of great importance.

Compared to other examination methods, US is a frontline imaging modality with advantages such as safety, non-invasiveness, cost-effectiveness, and convenience. Color Doppler flow imaging (CDFI) is commonly used during US examinations because of its ability to detect the motion of red blood cells as scatterers [8]. However, CDFI can only detect relatively fast flows (greater than 1 cm/s) in large vessels, making it unsuitable for imaging microvessel and slower flows within them [9]. In recent decades, contrast-enhanced US (CEUS) has been employed to diagnose a variety of diseases because it can significantly enhance the US contrast echo of the bloodstream. CEUS allows real-time visualization of the blood microcirculation within prostate lesion [10], and the analysis of CEUS parameters enables quantitative evaluation of microcirculation characteristics [11].

The imaging resolution of CEUS remains constrained by the diffraction limit [12], rendering it incapable of directly visualizing microvessel characteristics and insufficient for detecting flow velocity within microvessel. Inspired by optical super-resolution imaging (SRI) [13], US SRI technique overcomes the traditional diffraction resolution limit, which is unattainable with conventional US imaging [14]. US SRI technology, also known as US localization microscopy (ULM), effectively combines CEUS, high frame rate (HiFR) imaging, advanced clutter filtering technology, and innovative centroid localization methods [15, 16]. The notable feature of ULM is its ability to generate super-resolved flow velocity information within the microvessel by detecting and tracking the microbubbles (MBs), offering a non-invasive assessment of tissue blood flow at the microscopic level.

The US SRI has demonstrated the ability to display and quantify tumor microvessel with unprecedented resolution, as evidenced by studies ranging from animal tumor models in vivo [17] to human body [18, 19]. However, the feasibility of using US SRI to assess microvessel in human prostate lesion remains unknown. Consequently, this study aimed to explore the feasibility and clinical value of assessing microvessel characteristics and related parameters of prostate lesion using US SRI, and to assist in the differential diagnosis of prostate lesion using an innovative approach.

## Materials and methods

### Participants

**Fig. 1:**
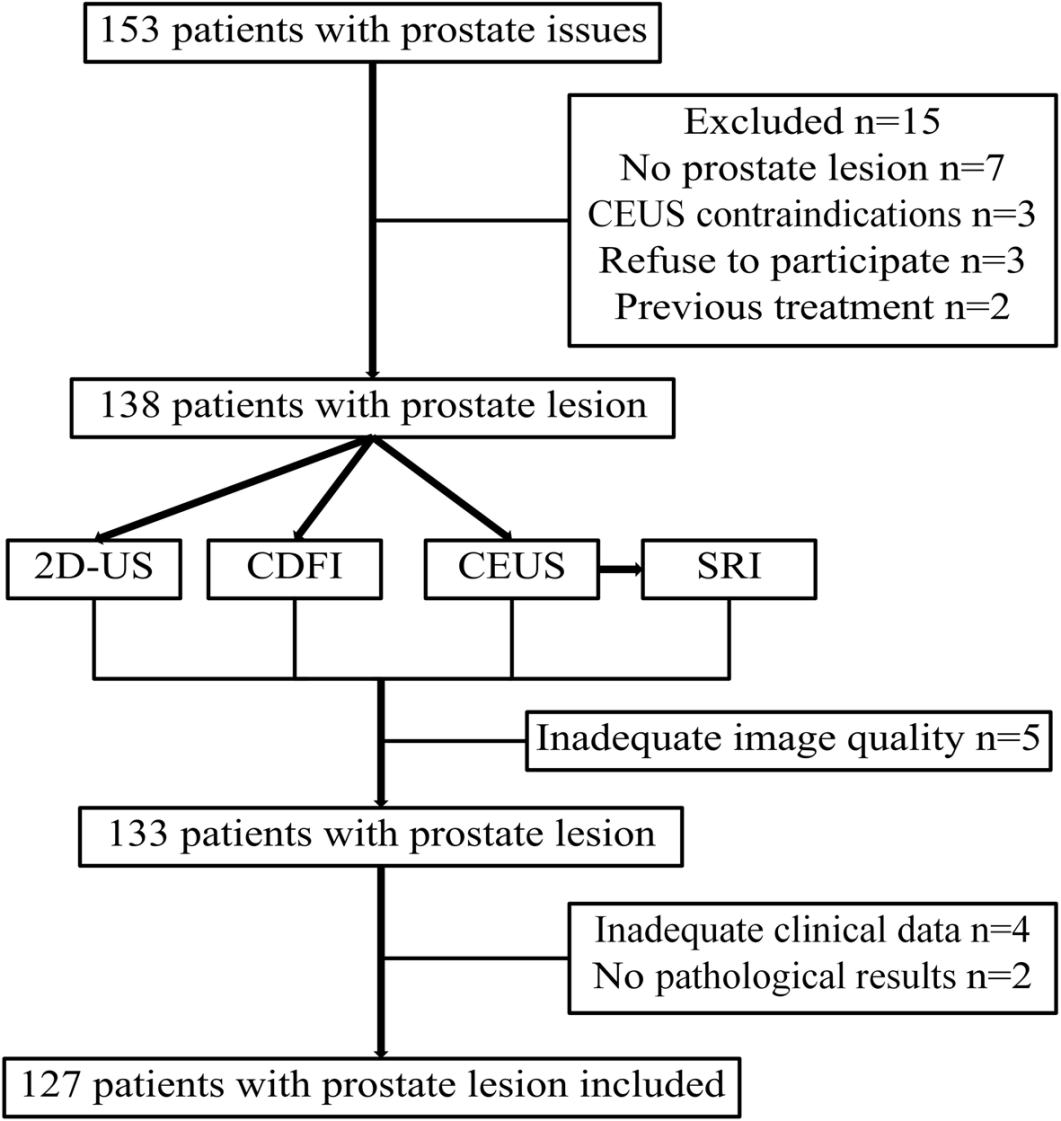
Flow diagram of patient enrollment and classification

This study involved male patients with prostate issues who visited Renmin Hospital of Wuhan University between November 2023 and June 2024. The inclusion criteria were as follows: age > 18 years, presence of prostate lesion detected by transrectal US (TRUS), clear 2D-US imaging, and pathological confirmation of the prostate lesion obtained via TRUS-guided biopsy. The exclusion criteria included contraindications to contrast agents or biopsy, prior tumor-related treatment before the examination, inadequate image quality for software analysis, and insufficient clinical data. Finally, 127 patients with prostate lesion were included in this exploratory study (Fig. 1). The study was approved by the Clinical Research Ethics Committee of Renmin Hospital of Wuhan University, and all participants provided informed consent.

### US images acquisition

All patients underwent TRUS and CEUS using a US platform (Resona R9S, Mindray Bio-Medical Electronics Co. Ltd., Shenzhen, China) equipped with an ELC13-4U convex linear dual-array probe. The dynamic range, gain, and depth settings were adjusted according to the specifications of the US machine. During the examination, patients were placed in the left lateral position. The prostate was examined by using B-mode and color Doppler flow imaging (CDFI) in convex array mode. If a prostate lesion was detected, B-mode and CDFI images were also collected.

### CEUS images acquisition

Under convex array mode, real time dual-mode imaging (B-mode and CEUS) was applied to the prostate lesion of patients using a mechanical index (MI) of 0.09 and frequency of 5.0 MHz. For CEUS, SonoVue solution (2.5 mL) was rapidly injected into the elbow vein, followed by 5 mL of normal saline. During the CEUS procedure, patients were instructed to maintain a stable position, and the probe was kept steady. Continuous dynamic images were collected for 2 min, with all images saved for further analysis. The prostate lesion was designated as the region of interest (ROI) for analysis using the US platform’s software, which provided the following time-intensity curve (TIC) parameters: arrival time (AT), rising time (RT), time to peak (TTP), peak intensity (PKI), falling time (FT), mean transit time (MTT), ascending slope (AS), descending slope (DS), D/A slope ratio (SR), and area under the TIC (AUC). The analysis was repeated three times and the average values of the perfusion parameters were calculated.

### US SRI imaging processing

Fifteen minutes after the initial CEUS, the contrast agent in the patient’s prostate had nearly completely dissipated. The probe was then switched to linear array mode, clearly display the lesion. Subsequently, injection of 0.5 mL of MBs was administered through the elbow vein for a second imaging session under the HiFR CEUS module, with MI of 0.08 and frequency of 3.9 MHz. Continuous dynamic images were collected for 2 min, and all images were saved for further analysis.

The HiFR CEUS data output from the US equipment was utilized for SRI processing, which was performed offline using MATLAB (MathWorks Inc., Natick, MA, USA). Each frame in the dataset underwent singular value decomposition (SVD) processing to remove clutter and background signals. Following SVD processing, each frame underwent super-localization processing. Specifically, an image pixel value threshold was set to eliminate noise signals and isolate MBs signals. The area (A), intensity (I), and shape/eccentricity (E) of each MBs signal were measured. These indices were used to filter out non-MBs signals and artifacts. The coordinates of spatially isolated signals were determined using the “centroid” method, which calculated the intensity-weighted center of the signals. The localizations from all frames were aggregated to form the final SRI image.

The tracked MBs area was defined as the total microvasculature observed on the SRI. Microvessel density (MVD) was calculated by dividing the tracked MBs area by the area of the region of interest (ROI), which was manually delineated in MATLAB to correspond to the outlines of the prostate lesion on both the B-mode image and the associated super-resolved velocity map. Specifically, each MBs signal detected in frame K and each signal in frame K + 1 was identified within a search window. Given the frame rate of 80 Hz, the maximum search window was set at 700 micrometers, accommodating flow rates of up to 20 mm/s. If the maximum normalized cross-correlation of each signal in frame K exceeded an empirically set threshold of 0.9, a corresponding signal in frame K + 1 was identified. The tracking method utilized the best-matched bubble signals within the specified ROI between consecutive frames to calculate additional parameters, including microvessel diameter (D), microvessel velocity (V), microvessel tortuosity (T), and fractal number (FN). The average value of each parameter was then obtained.

### Statistical analysis

Continuous variables with a normal distribution were reported as mean ± standard deviation. Comparisons between two variables were conducted using the independent-sample t-test or the Mann-Whitney U test, as appropriate. A *P*-value of less than 0.05 was considered statistically significant.

## Results

### Clinical information

TRUS-guided core-needle biopsy was performed for 127 patients with prostate lesion, and pathological results were obtained. Based on these results, 78 patients were classified into the benign group, and 49 patients were classified into the malignant group. As showed in Table 1, no significant differences were observed in age and prostate size between the two groups (*P* > 0.05). However, prostate acid phosphatase (PAP), total prostate-specific antigen (tPSA), free prostate-specific antigen (fPSA), and the fPSA/tPSA ratio were all significantly higher in the malignant group compared to the benign group, with these differences being statistically significant (*P* < 0.01).

**Table 1:** Summary of clinical information of all patients

	benign (*n* = 78)	malignant (*n* = 49)	*P* value
Age (year)	69.93 ± 5.17	70.32 ± 8.44	0.825
Prostate tumor markers			
PAP (µmol/L)	2.18 ± 0.92	13.94 ± 10.81	<0.001
tPSA (µmol/L)	12.33 ± 9.97	127.64 ± 118.25	<0.001
fPSA (µmol/L)	2.66 ± 2.31	33.85 ± 27.93	< 0.001
f/tPSA (%)	0.18 ± 0.08	0.25 ± 0.13	0.004
Prostate size			
Left right diameter (mm)	53.13 ± 4.86	51.34 ± 5.26	0.081
Upper and lower diameter (mm)	49.17 ± 5.52	48.28 ± 5.93	0.538
Front and rear diameter (mm)	41.42 ± 7.53	40.37 ± 6.91	0.310
Volume (mL)	52.39 ± 7.52	49.85 ± 6.74	0.149

### CEUS of prostate lesion

**Fig. 2:**
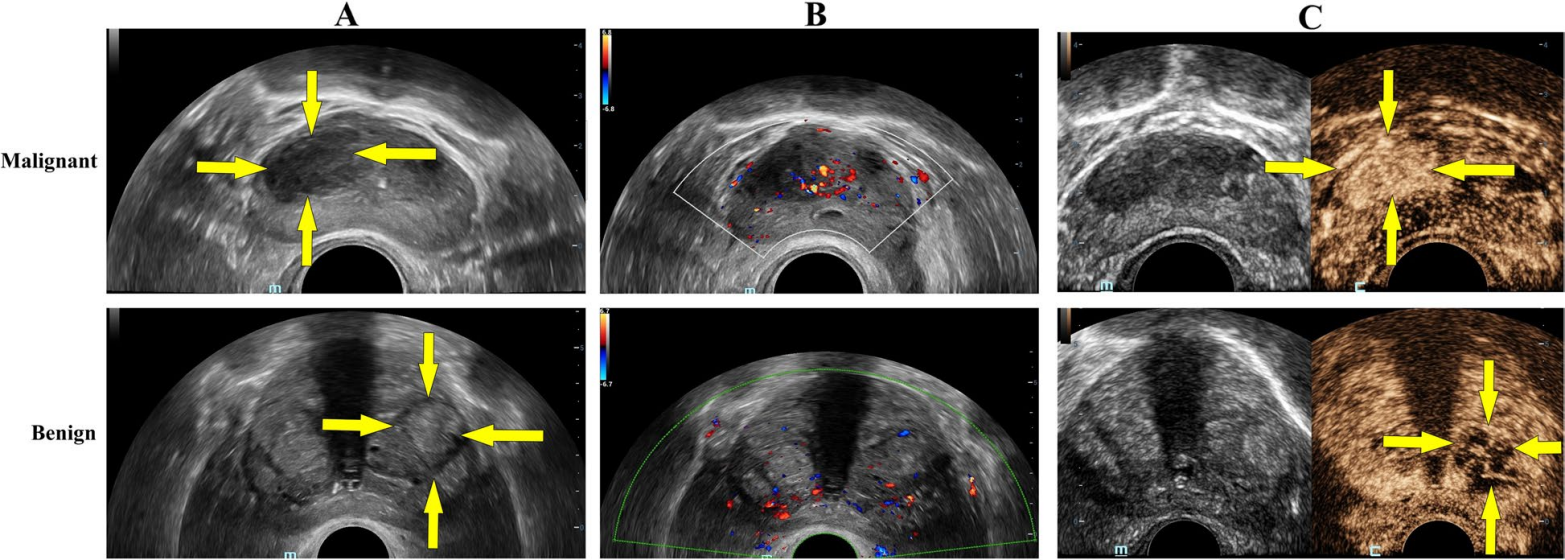
The imaging of prostate lesion under convex array mode. **A** 2D-US of prostate lesion, as indicated by the yellow arrow (**B**) CDFI of prostate lesion (**C**) CEUS performance of prostate lesion at peak time, as indicated by the yellow arrow

Following the injection of the MBs solution, the CEUS images displayed a hypo-enhancement pattern in benign prostate lesion, whereas malignant lesion exhibited a hyper-enhancement pattern (Fig. 2C). Additionally, the TIC parameters of CEUS revealed that the PKI, AS, DS, and AUC were significantly higher in the malignant group than in the benign group (*P* < 0.001), whereas the RT, TTP and FT in the malignant group were significantly lower (*P* < 0.001). However, AT, MTT, and SR did not significantly differ between the two groups (*P* > 0.05) (Table 2).

**Table 2:** Comparison of prostate lesion CEUS parameters

	benign (*n* = 78)	malignant (*n* = 49)	*P* value
AT (s)	14.63 ± 2.53	14.71 ± 2.84	0.328
RT (s)	11.22 ± 1.37	9.69 ± 1.41	< 0.001
TTP (s)	25.86 ± 2.98	23.18 ± 3.26	< 0.001
PKI (dB)	27.76 ± 5.31	33.58 ± 5.98	< 0.001
FT (s)	60.78 ± 7.24	53.97 ± 9.31	< 0.001
MTT (s)	63.07 ± 4.04	62.86 ± 4.77	0.256
AS	2.35 ± 0.47	2.49 ± 0.64	< 0.001
DS	0.21 ± 0.04	0.24 ± 0.06	< 0.001
SR	0.10 ± 0.03	0.11 ± 0.04	0.121
AUC (dB∙s)	1843.91 ± 435.61	2033.07 ± 718.19	< 0.001

### US SRI and parameters

**Fig. 3:**
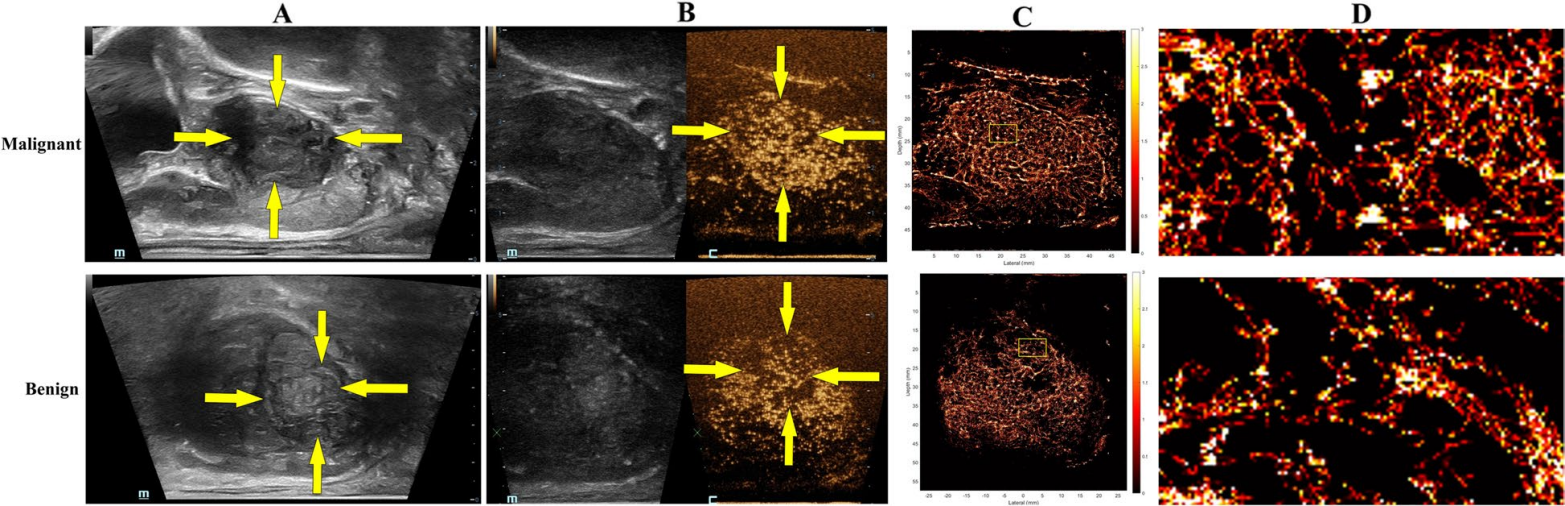
The imaging of prostate lesion under linear array mode. **A** 2D-US of prostate lesion, as indicated by the yellow arrow (**B**) HiFR-CEUS of prostate lesion, as indicated by the yellow arrow (**C**) SRI of prostate lesion (**D**) Zoomed-in sections as the yellow box indicated in C

**Fig. 4:**
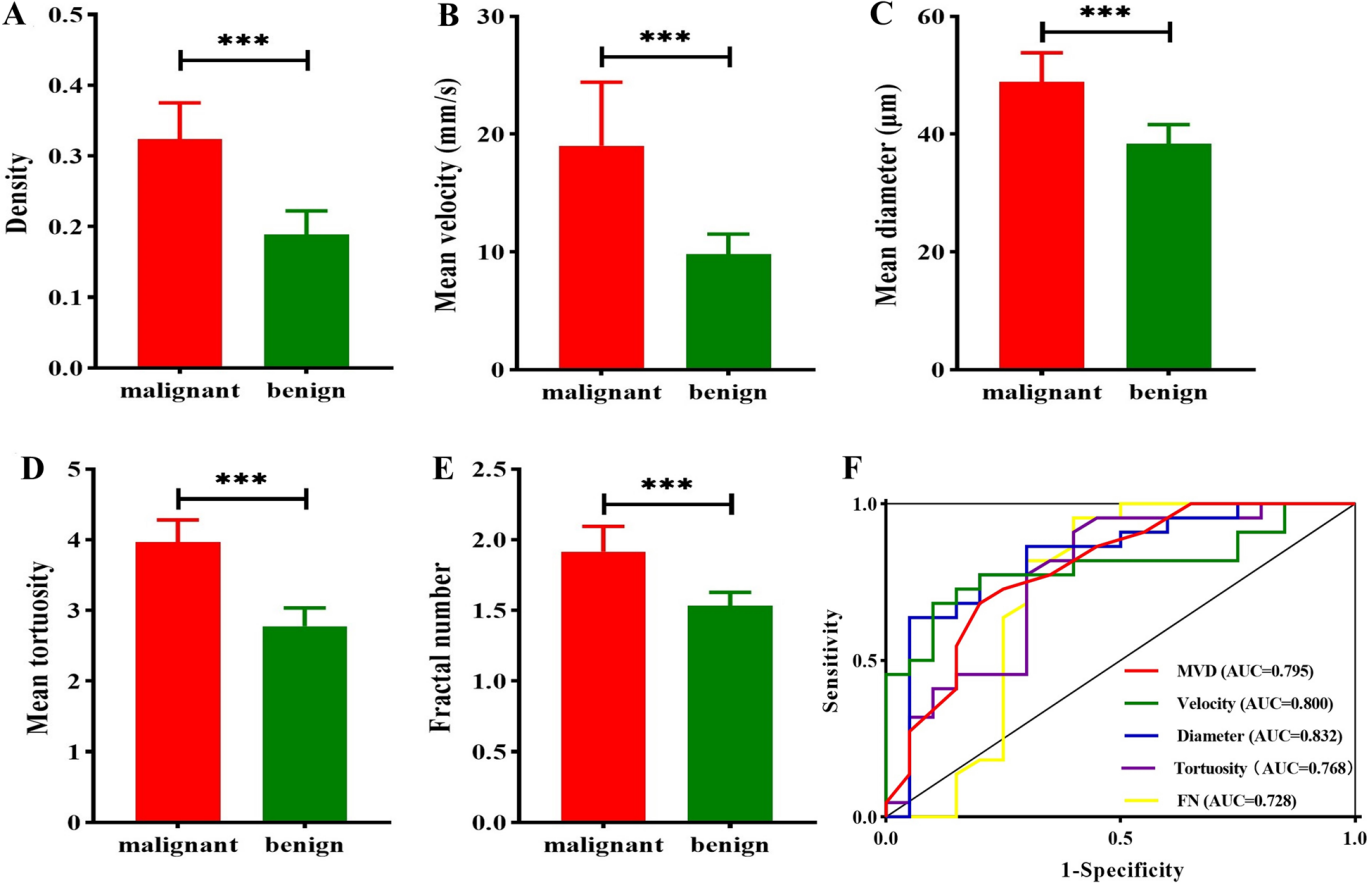
SRI parameters of prostate lesion. **A**-**E** Comparison of malignant and benign lesion of MVD, V, D, T, FN (**F**) AUC of the SRI parameters

US SRI revealed that the microvessel distribution exhibited a complex pattern, differing from the more restrictive patterns observed with CDFI (Fig. 2B) and CEUS in prostate lesion. Additionally, SRI significantly enhanced spatial resolution, achieving sub-100 micrometer precision for diameter (Fig. 3D). And the D was (38.35 ± 3.26) µm in the benign group, respectively (48.89 ± 4.93) µm in the malignant group (Fig. 4C). Furthermore, the D performed the best diagnostic value, with AUC value of 0.832 (95% confidence interval [CI]: 0.685–0.929), a sensitivity of 85.7% (95% CI: 62.1–96.8%), and a specificity of 63.6% (95% CI: 40.7–82.8%) (Fig. 4F).

Similarly, other SRI parameters in the malignant group were significantly higher than those in the benign group. MVD of malignant group (0.32 ± 0.05) was higher than that of benign group (0.19 ± 0.03) (Fig. 4A), with AUC value of 0.795 (95% CI: 0.643–0.904) (Figs. 3C and 4F). The T and V also showed great diagnostic value, with the AUC value of 0.768 (95% CI: 0.612–0.884) and AUC of 0.800 (95% CI: 0.648–0.907) (Fig. 4F). The FN showed minimal diagnostic value, with AUC of 0.728 (95% CI:0.569–0.854) (Fig. 4F).

**Fig. 5:**
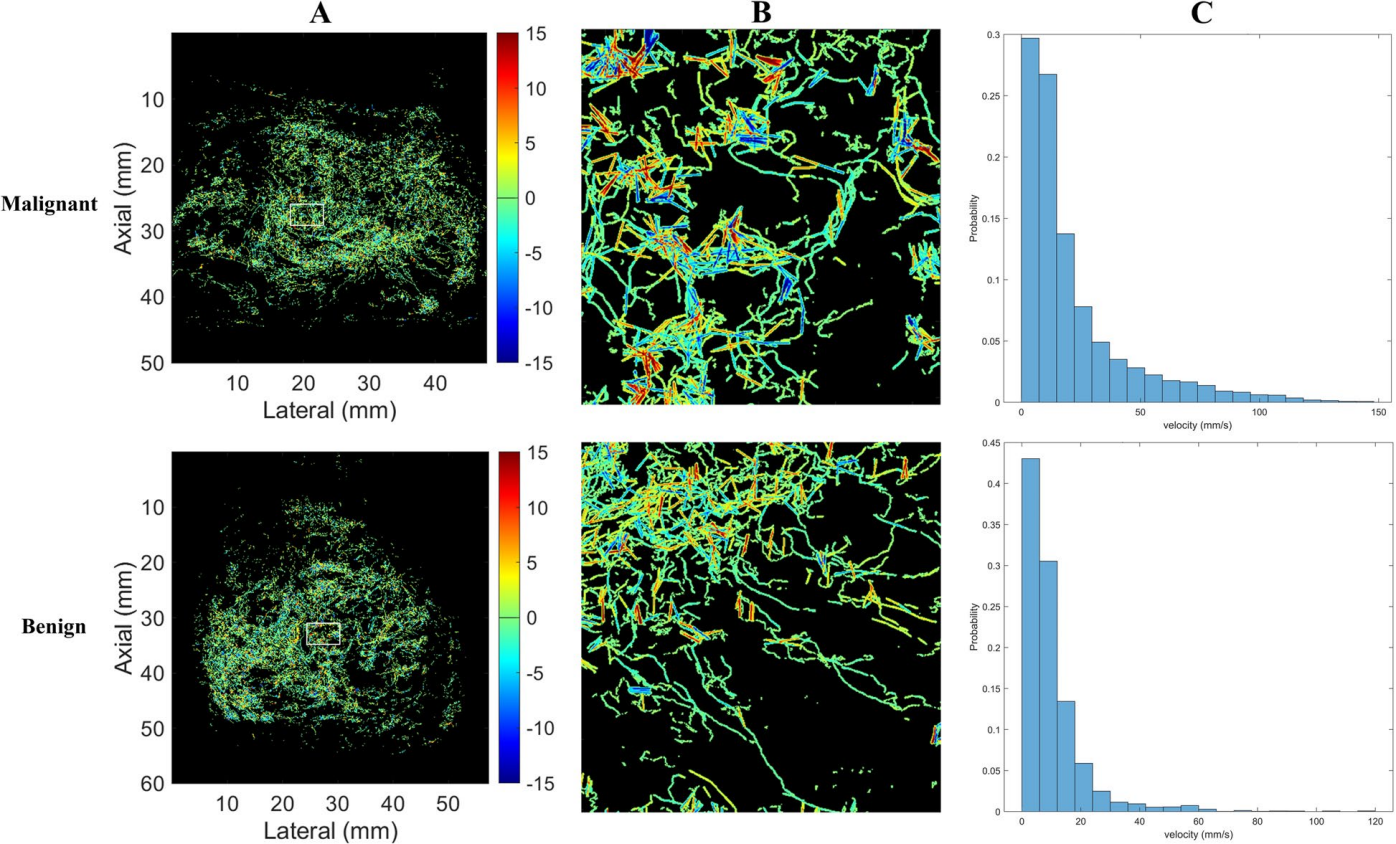
The velocity of prostate lesion. **A** SRVM of malignant and benign prostate lesion (**B**) Zoomed-in sections as the white box indicated in A (**C**) Velocity profile of malignant and benign prostate lesion

Similar to CDFI, the red and blue colors represented the relatively high flow rates in opposite directions and the yellow color represented relatively low flow rates in the super-resolved velocity map (SRVM). The V was (9.82 ± 1.69) mm/s of benign lesion, significantly lower than the malignant group of (19.02 ± 5.39) mm/s (Fig. 4B). And the velocity profile showed that the value range of malignant lesion was greater than benign (Fig. 5C), furthermore SRVM intuitively displays the V was greater in the malignant lesion (Fig. 5A and B), which demonstrates SRVM can show more information on microvascular flow velocity than CDFI.

## Discussion

Tumor angiogenesis serves as the physiological basis for tumor cell growth and metastasis [20], however conventional examination methods cannot effectively evaluate microvessel characteristics. CEUS has been widely utilized in the clinical diagnosis of tumors because MBs significantly enhance the acquisition of US signals through backscattering. Notably, the MBs can freely traverse tumor microcirculation, making it easier to detect microvessel [10, 11]. CEUS can display the microvascular perfusion of prostate lesion in real time [21], while it cannot visually depict the distribution of microvessel within the prostate lesion. Achieving non-invasive and sensitive detection of prostate lesion remains a significant challenge.

The emerging technique of super-resolution imaging (SRI) enables rapid image acquisition and tracking of MBs trajectories [22] by automatically generating microvessel images of living tissues through software. The MATLAB software used in this study has previously been applied to the analysis of microvessel structures in breast and thyroid lesions [19, 23]. This software can collect more than 1000 CEUS images at an average frame rate of 80 Hz, facilitating the tracking and recording of MBs trajectories and providing crucial support for microvessel assessment. Furthermore, it can quantify the characteristics of microvessel [24].

MVD is often regarded as a surrogate marker of angiogenesis in tumors, a risk factor for metastasis, and a predictor of poor prognosis in cancer patients [25, 26]. Previous pathological examinations of prostate specimens have shown that the MVD in malignant prostate lesion is significantly higher than that in surrounding normal tissues and benign lesion [7]. CEUS parameter analysis can reflect the microvascular perfusion characteristics of prostate lesion [27], and some parameters correlate highly with MVD. However, existing imaging methods are unable to effectively evaluate microvascular morphological characteristics. In this study, the MVD in the malignant group (0.32 ± 0.05) was also significantly higher than in the benign group (0.19 ± 0.03). Compared to benign lesion, SRI can also clearly demonstrate that the MVD in malignant lesion is significantly higher. Therefore, SRI technology can non-invasively evaluate the MVD of prostate lesion.

Tadayyon et al. evaluated the characteristics of MVD, size, and the percentage of irregular vessels in 62 PCa specimens [7]. They found that these characteristics were positively correlated with the gleason score (GS), suggesting that microvascular morphological characteristics can indirectly reflect the biological behavior of tumors. Killingsworth utilized transmission electron microscopy to thoroughly observe the neovascularization in PCa specimens with GS ranging from 4 to 9 [28], and the study revealed that the formation of new blood vessel begins with the growth of vascular endothelial cell buds, followed by a gradual widening of the lumen. Fully mature and functional neocapillaries typically have irregular contours and a zigzag pattern. As same as our study, SRI generated graph can intuitively find that T and FN of malignant lesion of prostate are significantly higher than those of benign lesion, and the measured values of the two also have statistical differences. The higher T represents the more irregular microvessels, and the larger FN reflects the more complex microvessel morphology.

Mucci et al. found a significant increase in the vessel diameter of resected specimens from 572 patients with PCa, but could not quantify the diameter of blood vessels [29]. In our study, the D of malignant group was (48.89 ± 4.93)um, and (38.35 ± 3.26) um with benign lesion. Furthermore, the V of malignant lesion (19.02 ± 5.39 mm/s) was faster than the benign (9.82 ± 1.69 mm/s). The significant differences are closely related to the greater demand for oxygen and nutrients of malignant tumors [30]. These findings are consistent with those of previous studies, suggesting that the angiogenic potential required for the progression of malignant prostate lesion is greater.

MVD, microvessel characteristics and microvessel maturity of malignant prostate lesion reflect tumor differentiation and prognosis to a certain extent. Accurate diagnosis and staging directly affect the choice of treatment options and curative effect. We will further study SRI technology to enhance the judgment efficiency of benign and malignant prostate lesion. At the same time, we will actively study the changes of MVD in patients with PCa who are not suitable for surgery, both before and after radiotherapy and chemotherapy, to introduce a non-invasive judgment method at the pathological level to the efficacy evaluation.

This study has some limitations. First, US SRI generation is time consuming and requires off-line processing. However, the US platform in our study has been used to achieve the visualization of microvessel in kidney, lymph and thyroid; therefore, we have reason to believe that it will soon be applied to prostate lesion. Second, the US SRI obtained in this study were two-dimensional imaging, therefore, the out of plane microvessel could not been revealed. A 3D SRI technique equipped with a 2D array probe is expected to overcome this problem in the future. Third, more cases will be collected to analyze the clinical utility of US SRI, and further investigation will be performed to compare the diagnostic efficacy of MRI and US SRI. Fourth, this was a preliminary study, and the current results cannot replace those of prostate biopsy. The continuous development of SRI will play an important role in the diagnosis of prostate lesion.

## Conclusions

US SRI technology is not only intuitive and non-invasive but also capable of quantifying the microvessel characteristics of prostate lesion with multiple parameters, offering an advancement in the differential diagnosis for PCa. Its ability to display and quantify microvessel with high precision could make US SRI a valuable tool in clinical practices.

## Data Availability

No datasets were generated or analysed during the current study.

## Abbreviations

PCa: Prostate Cancer
US: Ultrasound
SRI: Super-resolution imaging
TRUS: Transrectal US
CEUS: Contrast-enhanced US
AT: Arrival time
RT: Rising time
TTP: Time to peak
PKI: Peak intensity
FT: Falling time
MTT: Mean transit time
AS: Ascending slope
DS: Descending slope, AUC, area under the TIC
MVD: Microvessel density
D: Microvessel diameter
V: Microvessel velocity
T: Microvessel tortuosity
FN: Fractal number
MRI: Magnetic resonance imaging
PET/CT: Positron emission tomography/computed tomography
CDFI: Colour Doppler flow imaging
ULM: US localization microscopy
HiFR: High frame rate
MBs: Microbubbles
SVD: Singular value decomposition
ROI: Region of interest
MI: Mechanical index
A: Area
I: Intensity
E: Eccentricity
PAP: Prostate acid phosphatase
TIC: Time-intensity curve
tPSA: Total prostate-specific antigen
fPSA: Free prostate-specific antigen
SRVM: Super-resolved velocity map
GS: Gleason score
SR: D/A slope ratio
